# Exposure to strong irradiance exacerbates photoinhibition and suppresses N resorption during leaf senescence in shade-grown seedlings of fullmoon maple (*Acer japonicum*)

**DOI:** 10.3389/fpls.2022.1006413

**Published:** 2022-10-28

**Authors:** Mitsutoshi Kitao, Kenichi Yazaki, Hiroyuki Tobita, Evgenios Agathokleous, Junko Kishimoto, Atsushi Takabayashi, Ryouichi Tanaka

**Affiliations:** ^1^ Hokkaido Research Center, Forestry and Forest Products Research Institute, Sapporo, Japan; ^2^ Department of Plant Ecology, Forestry and Forest Products Research Institute, Tsukuba, Japan; ^3^ Department of Ecology, School of Applied Meteorology, Nanjing University of Information Science & Technology (NUIST), Nanjing, China; ^4^ Institute of Low Temperature Science, Hokkaido University, Sapporo, Japan

**Keywords:** inner-canopy leaves, sugar accumulation, holocanopy hypothesis, light attenuation, photooxidative stress

## Abstract

Leaves of fullmoon maple (*Acer japonicum*) turn brilliant red with anthocyanins synthesis in autumn. Based on field observations, autumn coloring mainly occurs in outer-canopy leaves exposed to sun, whereas inner-canopy leaves remain green for a certain longer period before finally turn yellowish red with a smaller amount of anthocyanins. Here, we hypothesized that outer-canopy leaves protect themselves against photooxidative stress *via* anthocyanins while simultaneously shading inner canopy leaves and protecting them from strong light (holocanopy hypothesis). To test this hypothesis, we investigated photoinhibition and leaf N content during autumn senescence in leaves of pot-grown seedlings of fullmoon maple either raised under shade (L0, ≈13% relative irradiance to open) or transferred to full sunlight conditions on 5^th^ (LH1), 12^th^ (LH2), or 18^th^ (LH3) Oct, 2021. Dry mass-based leaf N (N_mass_) in green leaves in shade-grown seedlings was ≈ 30 mg N g^-1^ in summer. N_mass_ in shed leaves (25^th^ Oct to 1^st^ Nov) was 11.1, 12.0, 14.6, and 10.1 mg N g^-1^ in L0, LH1, LH2, and LH3 conditions, respectively. Higher N_mass_ was observed in shed leaves in LH2, compared to other experimental conditions, suggesting an incomplete N resorption in LH2. F_v_/F_m_ after an overnight dark-adaptation, measured on 19^th^ Oct when leaf N was actively resorbed, ranked L0: 0.72 > LH3: 0.56 > LH1: 0.45 > LH2: 0.25. As decreased F_v_/F_m_ indicates photoinhibition, leaves in LH2 condition suffered the most severe photoinhibition. Leaf soluble sugar content decreased, but protein carbonylation increased with decreasing F_v_/F_m_ across shade-grown seedlings (L0, LH1, LH2, and LH3) on 19^th^ Oct, suggesting impaired photosynthetic carbon gain and possible membrane peroxidation induced by photooxidative stress, especially in LH2 condition with less N resorption efficiency. Although the impairment of N resorption seems to depend on the timing and intensity of strong light exposure, air temperature, and consequently the degree of photoinhibition, the photoprotective role of anthocyanins in outer-canopy leaves of fullmoon maple might also contribute to allow a safe N resorption in inner-canopy leaves by prolonged shading.

## Introduction

Autumn red coloring, a result of accumulation of anthocyanins, is considered to have a protective role against photooxidative stress under low temperature (photoprotection hypothesis) (2003; Feild et al., 2001; Hoch et al., 2001), where anthocyanins might act as light attenuators or antioxidants (Neill et al., 2002; Moustaka et al., 2020). Conversely, red color is also considered a signal against pest insects as a consequence of co-evolution (co-evolution hypothesis) (2021; Pena-Novas and Archetti, 2020). These two hypotheses are still debated (Renner and Zohner, 2019; Pena-Novas and Archetti, 2020; Hughes et al., 2022).

N resorption during autumn is an essential feature of deciduous trees for overwintering and growth in next spring (Hörtensteiner and Feller, 2002; Cooke and Weih, 2005; Niinemets and Tamm, 2005; Millard and Grelet, 2010; Tanaka and Tanaka, 2011; Tobita et al., 2021). According to the photoprotection hypothesis, anthocyanins are considered to contribute to efficient N resorption by means of preventing photooxidative stress (2020; Hoch et al., 2003; Renner and Zohner, 2019). Conversely, such a contribution of anthocyanins to efficient N resorption was not necessarily confirmed in other studies (2021; Feild et al., 2001; Pena-Novas and Archetti, 2020).

**Figure 1:**
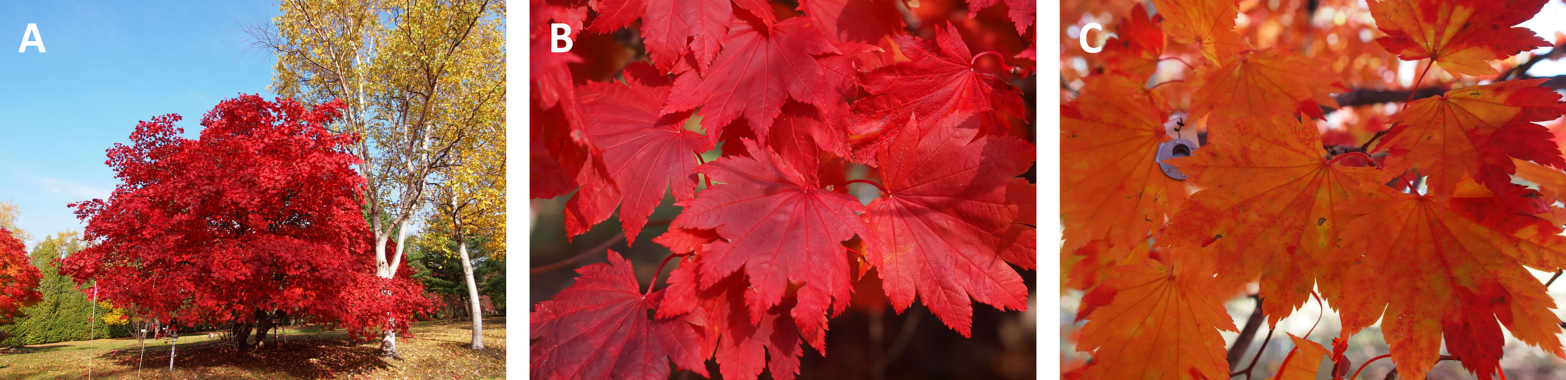
Autumn coloring in an adult tree of fullmoon maple (*Acer japonicum*) **(A)** in outer-canopy **(B)** and inner-canopy **(C)** leaves. Photographs were taken on 26 Oct, 2021, at the arboretum of Hokkaido Research Center, Forestry and Forest Products Research Institute, in Sapporo (43.0°N, 141.4°E; 180 m a.s.l.).

Fullmoon maple (*Acer japonicum*) is popular in Japan because of its beautiful brilliant red coloring in autumn. From field observations, autumn red coloring is predominant in leaves grown in the outer-canopy of fullmoon maple, whereas inner-canopy leaves remain green for a longer time and finally turn yellowish red with less anthocyanins (cf. Koike, 1990) (**Figure 1**). Leaves of fullmoon maple flush once in spring, and shed almost at the same time, irrespective of canopy position (Kikuzawa, 1983; Koike, 1990). The light environment within a canopy is substantially heterogenous. Leaves within a canopy acclimate to their growth light environment, where outer-canopy leaves have higher area-based leaf N content and photosynthetic capacity than inner-canopy leaves (2018b; Niinemets et al., 2004; Kitao et al., 2006; Niinemets, 2007). Regarding N resorption at the canopy level, heterogeneous light environments within the canopy should be taken into account.

Regarding species-specific autumn coloring, Koike (1990; 2004) summarized differences in autumn coloring among deciduous broad-leaf tree species with different successional traits. Late successional tree species, such as maple and cherry with flush type leaf development, change leaf color from the outer part of canopy. Early successional tree species, such as birch, poplar, and willow, show earlier leaf senescence in the inner canopy, with leaves generally turning yellow, even when the outer-canopy leaves are still green. As early successional species develop new leaves continuously, they have young leaves in the outer canopy and old leaves in the inner canopy. As for fullmoon maple, a typical anthocyanic species, classified as late successional deciduous broad-leaf tree species, outer-canopy leaves had higher amount of anthocyanins than inner-canopy leaves during leaf senescence, while leaf senescence was quite synchronized irrespective of the leaf position, based on the seasonal changes in dry mass-based leaf anthocyanins and N content (**Supplementary Figure 1**). Higher amount of anthocyanins in the outer leaves of fullmoon maple is consistent with the fact that an induction of anthocyanins synthesis requires high light intensities (Steyn et al., 2002).

Sugar accumulation might be a regulative signal of leaf senescence (Ono et al., 2001; Wingler et al., 2006). Anthocyanins are known to be synthesized from accumulated sugars in leaves during autumn (Stitt and Hurry, 2002; Hughes et al., 2022). Besides photoprotection, recently, a possible function of anthocyanins has been proposed as a sugar-buffer to moderate sugar feedback regulation, which prevents early sugar-mediated leaf senescence (Ono et al., 2001; Landi et al., 2015; Lo Piccolo et al., 2018; Lo Piccolo and Landi, 2021; Davies et al., 2022). Leaves in red-leafed *Prunus cerasifera* var. *pissardii* showed delayed leaf senescence with lower soluble sugar content than leaves in green-leafed *P. cerasifera* clone 29C (Lo Piccolo et al., 2018). The *Arabidopsis nla* (nitrogen limitation adaptation) mutant, which showed a lower production of anthocyanins than wild type under limiting N, showed earlier senescence (Peng et al., 2008). Furthermore, anthocyanins might prolong leaf longevity by delaying the progress of abscission layer in leaves of sugar maple (*Acer saccharum* Marsh.) (Schaberg et al., 2008).

Regarding the canopy-level response to photooxidative stress, outer-canopy leaves, acting as efficient light attenuators, might protect inner-canopy leaves against solar radiation under low temperature during leaf senescence. Photooxidative stress might directly interfere the cellular processes for N resorption, involved in membrane intactness (Hörtensteiner and Feller, 2002; Okumoto and Pilot, 2011), or indirectly reduce photosynthates necessary for protein breakdown and phloem loading of amino acids (Hörtensteiner and Feller, 2002; Cooke and Weih, 2005; Liu et al., 2008; Okumoto and Pilot, 2011). Exposure of inner-canopy leaves to strong light due to earlier shedding of outer-canopy leaves might cause photooxidative stress, leading to an insufficient N resorption.

Here, we assumed that higher amount of anthocyanins in the outer-canopy leaves of fullmoon maple might prevent early leaf senescence, leading to synchronized leaf senescence with inner-canopy leaves. Based on this assumption, we propose a novel hypothesis positing that outer-canopy leaves of fullmoon maple protect inner-canopy leaves from oxidative stress by shading, contributing to efficient N resorption in the inner-canopy leaves (holocanopy hypothesis) (holo: from the Greek word holos –όλoς-, meaning whole or entire). Validation of this hypothesis requires evidence that N resorption of inner-canopy leaves is protected by shading during leaf senescence. In other words, exposure of inner-canopy leaves to strong sunlight during autumn senescence (i.e. early shedding of outer-canopy leaves) might reduce N resorption *via* photooxidative stress. To test this hypothesis, we investigated photoinhibition and leaf N content during autumn senescence in leaves of shade-grown seedlings of fullmoon maple, transferred to the full sunlight condition on different dates during autumn senescence, simulating early shedding of outer-canopy leaves.

## Materials and methods

### Plant materials

Four-year-old bare-root seedlings of fullmoon maple (*Acer japonicum*) (≈ 40 cm in shoot height) were transplanted into 4-L plastic pots, filled with clay loam soil mixed with Kanuma pumice soil (1:1 in volume), at the end of April 2021. We added 40 g pot^-1^ of commonly-used fertilizer (Osmocote Exact Standard 15-9-11 +TE, HYPONeX Japan, Osaka, Japan). Twenty six seedlings were grown under natural light (H0), while the other 26 seedlings were grown in an experimental house (width: 2 m x length: 5 m x height: 2m) covered with a shade cloth (relative light irradiance, ≈ 13% to open) (L0). Leaves flushed within a few days after transplanting into the pots. We used leaves with the same leaf age, which flushed in spring.

**Figure 2:**
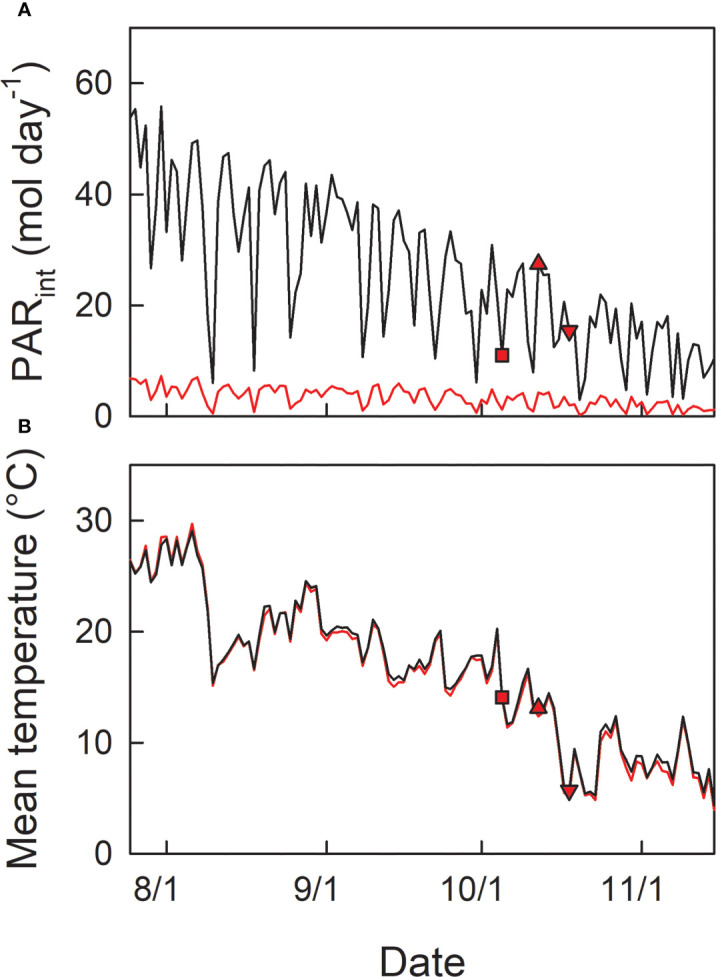
Seasonal changes in daily integrated photosynthetically active radiation (PAR_int_) **(A)**, and daily mean temperature **(B)** in open (black line) and shade (red line) conditions. Red symbols indicate the date of transfer of shade-grown seedlings to the open condition; LH1 (square): 5^th^ Oct, LH2 (triangle-up): 12^th^ Oct, and LH3 (triangle-down): 18^th^ Oct, 2021.

Seedlings were grown at the respective light conditions during the summertime. Then, seedlings grown under shade were transferred into open conditions (the same place where H0 plants were cultivated) in autumn, on 5^th^ Oct (LH1), 12^th^ Oct (LH2), or 18^th^ Oct (LH3), simulating early shedding of outer leaves in the canopy. Basically 4 seedlings were used as replicates for each experimental condition (treatment). Photosynthetically active radiance (PAR) and air temperature at open and shade conditions were monitored by photo-sensors (S-LIA-M003, Onset Computer Corporation, Bourne, MA, USA), and thermo-sensors (S-THB-M002, Onset Computer Corporation) placed in solar radiation shields (RS3, Onset Computer Corporation), combined with data loggers (H21-USB, Onset Computer Corporation) (**Figure 2**).

### Chlorophyll fluorescence measurements

F_v_/F_m_ = (F_m_-F_o_)/F_m_, was measured after an overnight dark-adaptation with a chlorophyll fluorometer (Mini-PAMII, Walz, Effeltrich, Germany) periodically from summer to autumn until the leaves shed. F_m_ is the maximum fluorescence level elicited by a pulse of saturating light (≈ 6000 µmol m^-2^ s^-1^), and F_o_ is the minimum fluorescence level. Although F_v_/F_m_ cannot be equated with the quantum efficiency of PSII photochemistry (Sipka et al., 2021), a decrease in F_v_/F_m_ might still be used as an empirical indicator of photoinhibition (Krause, 1994; Werner et al., 2002). One leaf per seedling was used for the measurement. Dark adaptation clips (DLC-8, Walz) were attached to leaves in the evening of the previous day for the measurements. We measured F_v_/F_m_ in the following morning after an overnight dark-adaptation. We started the measurements of F_v_/F_m_ on 2^nd^ Aug, 2021. Regarding shade-grown seedlings transferred into the open condition, we attached the dark adaptation clips in the evening of 4^th^, 11^th^, and 17^th^ Oct in LH1, LH2, and LH3 seedlings, respectively. After measuring F_v_/F_m_ on the following morning (5^th^, 12^th^, and 18^th^ Oct) to evaluate F_v_/F_m_ before transfer, seedlings were transferred to the open condition. We also measured F_v_/F_m_ 1 day after the transfer, specifically on 6^th^, 13^th^, and 19^th^ Oct, respectively. Then, we monitored F_v_/F_m_ at an ≈ 1-week interval.

### Leaf sugar, starch and nitrogen contents

Leaves used for F_v_/F_m_ were sampled immediately after the measurements. One third of a whole leaf was sampled for determination of leaf sugar, starch, and N contents. The rest two third was frozen with liquid nitrogen, and stored at −80°C until further analysis of protein carbonylation and leaf pigments, as described below. Regarding shed leaves, several leaves per seedling were sampled for determination of leaf N contents. Leaf samples for sugar, starch, and N analyses were dried at 70°C to constant weight in an electric oven. Sugars were extracted with 80% ethanol and determined by the phenol–sulfuric acid method (DuBois et al., 1956). Absorbance was measured at 490 nm using a spectrophotometer (AE-450N, ERMA Inc., Tokyo, Japan). Starch in the residue was solubilized by potassium hydroxide, and then digested to glucose with amyloglucosidase solution (A9228, Sigma, St. Louis, Mo., USA) (Kabeya et al., 2003). The digested glucose was determined by the mutarotase-glucose oxidase method (Wako Autokit Glucose (439-90901), FUJIFILM Wako Pure Chemical Industries, Ltd., Osaka, Japan). Absorbance was measured at 505 nm using a microplate photometer (SH-1200, CORONA ELECTRIC Co. Ltd., Ibaraki, Japan). Dry-mass-based leaf nitrogen content (N_mass_) was determined by a nitrogen carbon analyzer with oxygen circulating combustion system (SUMIGRAPH, NC 22F, Sumika Chem. Anal. Service, Osaka, Japan). We assumed that leaf N content of H0 and L0 seedlings sampled on 2^nd^ Aug, 20^th^ Aug, and 8^th^ Sept as leaf N content in green leaves. Efficiency of N resorption was calculated as: resorption efficiency = ([N in green leaves] – [N in shed leaves])/[N in green leaves] × 100 (Hoch et al., 2003).

### Analysis of protein carbonylation

Frozen samples as described above were ground to powder in liquid N_2_. Twenty mg of powdered sample were mixed with 200 μL of LDS buffer consisting of 50 mM Tris HCl pH7.5, 0.3M sucrose, 0.1M dithiothreitol and 2% (w/v) lithium dodecyl sulfate, and incubated at 75°C for 5 min. The total protein concentration was determined by using a XL-Bradford kit (Pharma Foods International Co. Ltd., Kyoto, Japan) according to the manufacturer’s instruction. Protein carbonyl concentration was determined by derivatization with 2,4-dinitrophenylhydrazine (DNPH), using protein carbonyl assay kit (ab17820, Abcam plc, Cambridge, UK) according to the manufacturer’s instruction with a slight modification. Specifically, protein extracts were diluted with the dilution buffer supplied by the assay kit so that the protein concentration was adjusted to 0.36 mg/ml. Ten μL of each sample was loaded onto a 14% (w/v) polyacrylamide gel and resolved by SDS-PAGE. The gel was blotted onto PVDF membrane. Carbonylated protein was reacted with a primary antibody against the dinitrophenyl moiety and the secondary anti-IgG antibody conjugated with horse radish peroxidase which were supplied by the kit (ab178020, abcam), and was detected with fluorescent dye (NEL104001EA, Western Lightning Plus-ECL, PerkinElmer, MA, USA) by a charge-coupled-device camera (LuminoGraph II, ATTO Corp., Tokyo, Japan). The signals from all bands were combined to estimate the total protein carbonylation of each sample. Amounts of protein carbonylation were expressed in arbitrary units.

### Analyses of leaf pigments

Pigments (chlorophyll a, b, violaxanthin, antheraxanthin, and zeaxanthin) were extracted from 20 mg of frozen leaf materials by homogenization in pre-cooled acetone at –30˚C, as described by Furukawa et al. (2019). Extracts were centrifuged for 5 min at 21,600 × *g* at 4°C, and the supernatant was analyzed by high performance liquid chromatography (HPLC) using a C18 column (YMC-Pack ODS-AL 250 mm in length, 4.6 mm in i.d.; YMC Co., Ltd, Kyoto, Japan). The sample was eluted with an isocratic flow of solvent A (100% methanol) for 17 min, followed by a linear gradient from solvent A to B (60% methanol, 20% ethanol, 20% hexane) in 6 min and with an isocratic flow of solvent B at a flow rate of 0.8 mL min^–1^. The eluates were monitored by an L-2450 photodiode array detector (HITACHI High Technologies Science Corporation, Tokyo, Japan).

Frozen samples were ground to powder using Multi-Beads Shocker (Yasui Kikai Corporation, Osaka, Japan). Approximately 10 mg of powdered sample were mixed with 1 ml of 3 M HCl: H_2_O: MeOH (1: 3: 16, v: v: v). Anthocyanins were extracted using Shake Master (Biomedical Science Corporation, Tokyo, Japan) and 2.3-mm diameter zirconia beads for 2 min, and then incubated at 4°C for 2 h (Junker and Ensminger, 2016). Subsequently, the extract was centrifuged at 15,000 × *g* and 4°C for 5 min. The absorbance of the supernatant was determined at 524 and 653 nm with a spectrometer. Anthocyanin concentration was calculated as (A_524_ − 0.24A_653_)/33,000 [mmol mL^-1^], using a molar extinction coefficient of 33,000 M^−1^ cm^−1^ (Gould et al., 2000), and corrected for the interference by pheophytins (Murray and Hackett, 1991).

### Statistical analyses

One factorial ANOVA was employed to investigate the differences among the light treatments (H0, L0, LH1, LH2, and LH3) in F_v_/F_m_, leaf N and resorption efficiency, leaf sugar and starch contents, and leaf pigment contents on each date (R Core Team and R Development Core Team, 2020). When there was at least one significant difference among light treatments based on the ANOVA, Tukey-Kramer *post-hoc* test followed. We applied linear regression analyses to investigate the responses of 1) leaf sugar content, 2) leaf starch content, and 3) protein carbonylation to F_v_/F_m_. The level of significance was 0.05.

## Results

**Figure 3:**
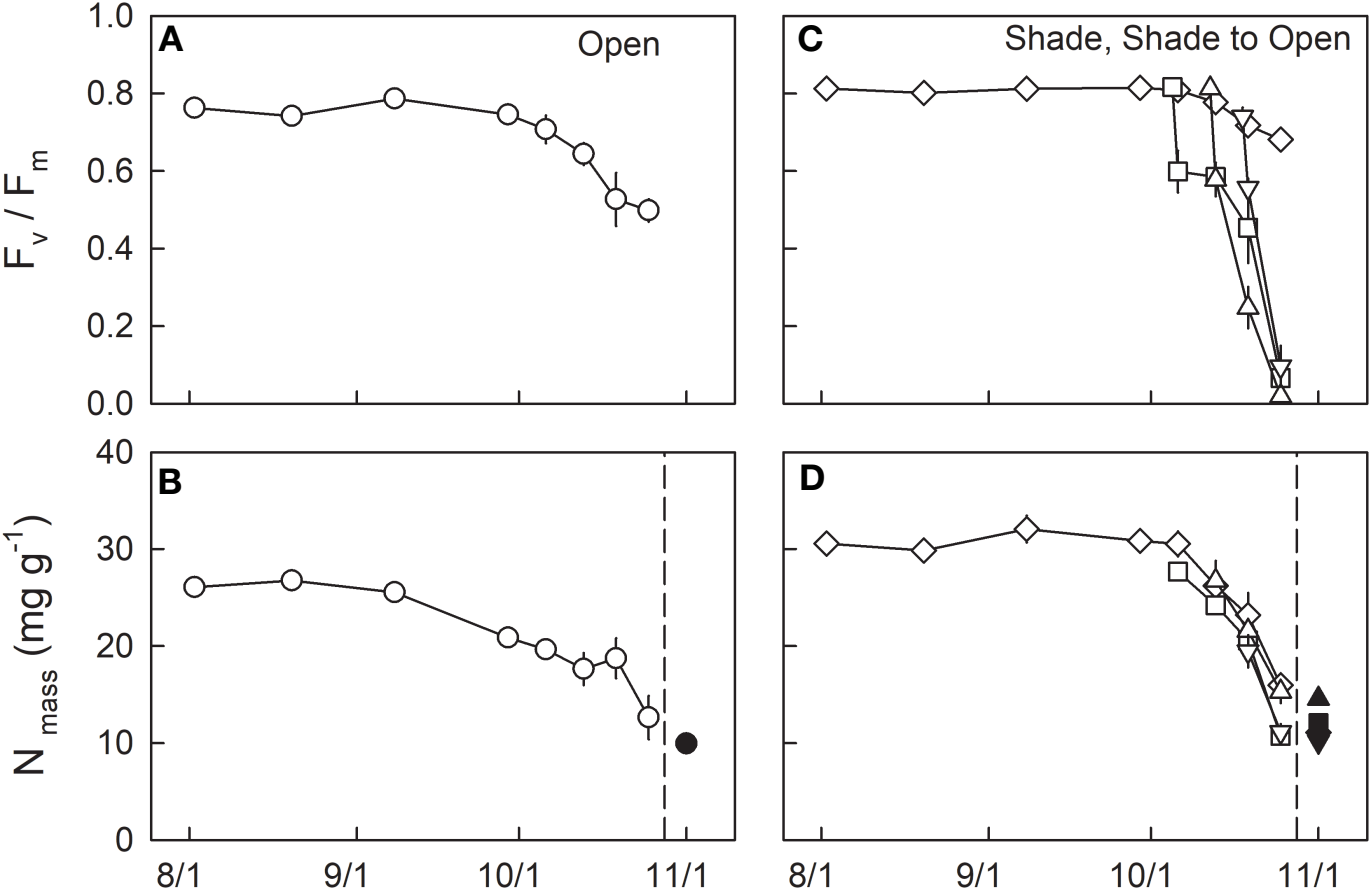
Seasonal changes in F_v_/F_m_ **(A, C)** and dry mass-based leaf N **(B, D)** in leaves of fullmoon maple grown under open (H0: circle), shade (L0: diamond), and shade to open (LH1: square, LH2: triangle up, and LH3: triangle down) conditions. Seedlings grown under shade were transferred to open conditions on 5^th^ (LH1: square), 12^th^ (LH2: triangle up), and 18^th^ (LH3: triangle down) of October. Open symbols indicate attached leaves (n = 3 to 6), whereas closed symbols indicate pooled data for shed leaves collected from 25 October to 1 November (n = 9 to 22). Values are means ± se. Detailed results of statistical analyses are shown in **Tables 1**, **2**.

F_v_/F_m_ during the summertime from the beginning of August to the end of September 2021 was relatively constant at ≈ 0.76 and 0.81 in H0, and L0 seedlings, respectively (**Figures 3A, C**; **Table 1**). From the beginning of October, F_v_/F_m_ started to decrease and reached 0.5 in H0 and 0.68 in L0 seedlings by the end of October. The daily-integrated PAR (PAR_int_) of the initial day of transfer was 11.0, 27.5, and 15.5 mol m^-2^ day^-1^ for LH1, LH2, and LH3 seedlings, respectively (**Figure 2A**). Daily mean temperature of the initial day of transfer was 14.1, 13.1, and 5.7°C for LH1, LH2, and LH3 seedlings, respectively (**Figure 2B**). F_v_/F_m_ in LH1, LH2, and LH3 seedlings decreased from 0.82, 0.81, and 0.74 to 0.60, 0.58, and 0.56, respectively, one day after the transfer. Although F_v_/F_m_ decreased linearly until 25^th^ Oct in LH2 and LH3 seedlings, it stopped decreasing temporarily from 6^th^ to 13^th^ Oct in LH1 seedlings, but then decreased again until 25^th^ Oct (**Figure 3C**; **Table 1**). The daily mean temperature from 6^th^ to 13^th^ Oct kept relatively high, around 15°C, with relatively high PAR_int_ (> 20 mol m^-2^ day^-1^ for 4 days, not including 13^th^ Oct). However, it suddenly dropped from 16^th^ to 23^th^ Oct, reaching a minimum value of 5.2°C (**Figures 2A, B**). On 19^th^ Oct, during the course of N resorption (cf. **Figure 3D**), LH2 seedlings showed the lowest F_v_/F_m_, compared with F_v_/F_m_ in L0, LH1, and LH3 seedlings (**Figure 3C**; **Table 1**).

**Table 1:** Seasonal change in F_v_/F_m_ after an overnight dark-adaptation in leaves of fullmoon maple seedlings grown under different light conditions.

Date	Open	Shade				
	H0	L0	LH1	LH2	LH3	F-statistics
Aug 2	0.76 ± 0.01 ^b^	0.81 ± 0.00 ^a^	―	―	―	28.0^**^ (F_1,6_)
Aug 20	0.74 ± 0.01 ^b^	0.80 ± 0.01 ^a^	―	―	―	21.9^**^ (F_1,6_)
Sep 8	0.79 ± 0.02 ^b^	0.81 ± 0.01 ^a^	―	―	―	8.03^*^ (F_1,6_)
Sep 29	0.75 ± 0.02 ^b^	0.81 ± 0.01 ^a^	―	―	―	12.6^*^ (F_1,6_)
Oct 5	―	―	*0.82 ± 0.01*	―	―	
Oct 6	0.71 ± 0.04 ^ab^	0.81 ± 0.01 ^a^	0.60 ± 0.05 ^b^	―	―	8.49^*^ (F_2,8_)
Oct 12	―	―	―	*0.81 ± 0.01*	―	
Oct 13	0.64 ± 0.03 ^b^	0.78 ± 0.01 ^a^	0.59 ± 0.02 ^b^	0.58 ± 0.04 ^b^	―	9.85^**^ (F_3,11_)
Oct 18	―	―	―	―	*0.74 ± 0.02*	
Oct 19	0.53 ± 0.07 ^ab^	0.72 ± 0.03 ^a^	0.45 ± 0.09 ^bc^	0.25 ± 0.05 ^c^	0.56 ± 0.02 ^ab^	13.1^***^ (F_4,17_)
Oct 25	0.50 ± 0.03 ^b^	0.68 ± 0.01 ^a^	0.07 ± 0.04 ^c^	0.02 ± 0.01 ^c^	0.10 ± 0.05 ^c^	76.4^***^ (F_4,15_)

H0: open condition, L0: shade condition (relative irradiance of ≈ 13% of open condition) throughout the experimental period, LH1: transfer from shade to open on Oct 5, LH2: transfer from shade to open on Oct 12, and LH3: transfer from shade to open on Oct 18. Values are means ± se; n= 3 to 6 for attached leaves. Italic indicates F_v_/F_m_ in shade-grown leaves, measured in the morning of the day of transfer. * denotes significant effect at P ≤ 0.05, ** P ≤ 0.01, and *** P ≤ 0.001. ns indicates no significant effect. Different letters indicate significant differences among means of the light treatments on the same date at P < 0.05.

The mean value of dry-mass-based leaf N content (N_mass_) in green leaves (2^nd^ Aug to 8^th^ Sep) was significantly lower in H0 seedlings (26.1 ± 0.1 mg g^-1^, n=12) than in shade-grown seedlings (L0: 30.8 ± 0.7 mg g^-1^, n=12), based on the student’s t test (*P* < 0.001). N_mass_ in H0 seedlings decreased from the end of September, whereas that in shade-grown seedlings (L0) decreased from the beginning of October (**Figures 3B, D**). Among shade-grown seedlings, L0 and LH2 seedlings had higher N_mass_ compared with LH1 and LH3 just before leaf shedding on 25 Oct (**Figure 3D**; **Table 2**). Conversely, significantly higher N_mass_ was observed in shed leaves of LH2 seedlings than those in the other treatments. Accordingly, resorption efficiency was significantly lower in LH2 seedlings (52.8%) when compared with that in L0 seedlings (64.0%).

**Table 2:** Seasonal change in dry-mass based leaf N content (N_mass_, mg g^-1^) and resorption efficiency (%) in leaves of fullmoon maple seedlings grown under different light conditions.

Date	Open	Shade				
	H0	L0	LH1	LH2	LH3	F-statistics
Aug 2	26.1 ± 0.6 ^b^	30.6 ± 0.9 ^a^	―	―	―	17.0^**^ (F_1,6_)
Aug 20	26.8 ± 0.4 ^b^	29.9 ± 1.2 ^a^	―	―	―	6.01^*^ (F_1,6_)
Sep 8	25.5 ± 0.8 ^b^	32.1 ± 1.4 ^a^	―	―	―	17.0^**^ (F_1,6_)
Sep 29	20.9 ± 0.4 ^b^	30.8 ± 1.1 ^a^	―	―	―	76.0^***^ (F_1,6_)
Oct 6	19.7 ± 0.8 ^b^	30.5 ± 1.3 ^a^	27.7 ± 0.7 ^a^	―	―	32.5^***^ (F_2,8_)
Oct 13	17.6 ± 1.7 ^b^	27.2 ± 1.9 ^a^	24.2 ± 0.3 ^ab^	26.7 ± 0.5 ^a^	―	6.69^**^ (F_3,11_)
Oct 19	18.7 ± 2.1	23.2 ± 2.3	20.6 ± 1.0	21.6 ± 0.6	19.5 ± 1.7	0.97^ns^ (F_4,17_)
Oct 25	12.6 ± 2.3 ^ab^	16.0 ± 0.9 ^a^	10.7 ± 0.4 ^b^	15.3 ± 1.2 ^ab^	11.1 ± 0.8 ^b^	4.58^ns^ (F_4,15_)
Shed leaves	10.0 ± 1.0 ^b^	11.1 ± 0.4 ^b^	12.0 ± 0.6 ^b^	14.6 ± 0.6 ^a^	10.1 ± 0.2 ^b^	12.0^***^ (F_4,70_)
Resorption efficiency (%)	57.5 ± 8.4^a^	64.0 ± 1.3^a^	65.9 ± 1.9 ^a^	52.8 ± 1.9 ^b^	67.1 ± 0.8 ^a^	9.48^***^ (F_4,70_)

H0: open condition, L0: shade condition (relative irradiance of ≈ 13% of open condition) throughout the experimental period, LH1: transfer from shade to open on Oct 5, LH2: transfer from shade to open on Oct 12, and LH3: transfer from shade to open on Oct 18. Values are means ± se; n= 3 to 6 for attached leaves, sampled from Aug 2 to Oct 25, and n = 9 to 22 for shed leaves collected from Oct 25 to Nov 1. * denotes significant effect at P ≤ 0.05, ** P ≤ 0.01, and *** P ≤ 0.001. ns indicates no significant effect. Different letters indicate significant differences among means of the light treatments on the same date at P < 0.05.

**Figure 4:**
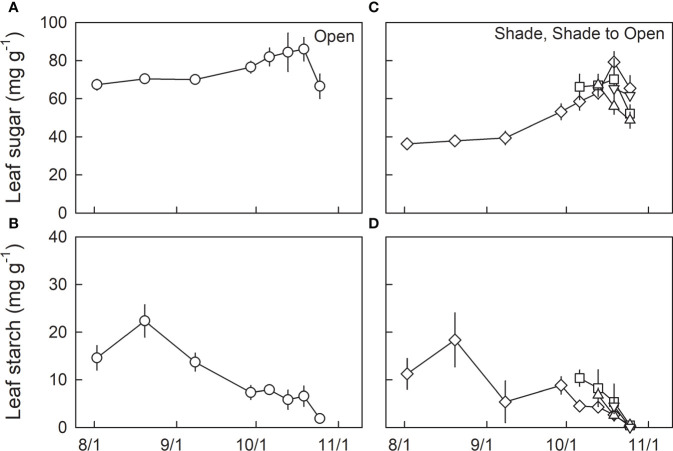
Seasonal changes in dry mass-based leaf sugar **(A, C)** and leaf starch content **(B, D)** in leaves of fullmoon maple grown under open (H0: circle), shade (L0: diamond), and shade to open (LH1: square, LH2: triangle up, and LH3: triangle down) conditions. Seedlings grown under shade were transferred to open conditions on 5^th^ (LH1: square), 12^th^ (LH2: triangle up), and 18^th^ (LH3: triangle down) of October (n = 3 to 6). Values are means ± se. Detailed results of statistical analyses are shown in **Tables 3**, **4**.

Leaf sugar content was relatively higher in H0 seedlings than in L0 seedlings during summertime (**Figures 4A, C**; **Table 3**). Leaf sugar content increased from late summer to autumn irrespective of light conditions, whereas L0 showed a greater increase than H0, resulting in no significant difference in leaf sugar content between H0 and L0 seedlings after 13^th^ Oct, which corresponds to the onset of N resorption (**Table 3**; cf. **Figure 3D**). Although no significant difference in leaf sugar content among shade-grown seedlings with different transfer timings (L0, LH1, LH2, and LH2) was detected, a wide variation in leaf sugar content was observed on 19^th^ Oct, where a marginal difference between L0 and LH2 (*P*=0.09) was detected.

**Table 3:** Seasonal change in dry-mass based leaf sugar content (mg g^-1^) in leaves of fullmoon maple seedlings grown under different light conditions.

Date	Open	Shade				
	H0	L0	LH1	LH2	LH3	F-statistics
Aug 2	67.4 ± 2.9 ^a^	36.3 ± 1.6 ^b^	―	―	―	88.6^***^ (F_1,6_)
Aug 20	70.4 ± 1.6 ^a^	37.9 ± 2.8 ^b^	―	―	―	98.7^***^ (F_1,6_)
Sep 8	70.1 ± 1.8 ^a^	39.4 ± 3.6 ^b^	―	―	―	59.5^***^ (F_1,6_)
Sep 29	76.6 ± 3.1 ^a^	53.1 ± 4.2 ^b^	―	―	―	20.4^**^ (F_1,6_)
Oct 6	82.0 ± 4.7 ^a^	58.5 ± 4.5 ^b^	66.3 ± 6.7 ^ab^	―	―	5.82^*^ (F_2,8_)
Oct 13	84.4 ± 10.1	63.1 ± 3.3	67.1 ± 5.8	67.2 ± 3.5	―	2.32^ns^ (F_3,11_)
Oct 19	86.0 ± 6.2 ^a^	79.3 ± 5.5 ^ab^	70.3 ± 10.7 ^ab^	56.0 ± 4.2 ^b^	65.4 ± 1.6 ^ab^	3.33^*^ (F_4,17_)
Oct 25	66.5 ± 6.4	65.5 ± 6.7	52.2 ± 4.6	48.8 ± 4.2	61.7 ± 0.3	1.44^ns^ (F_4,15_)

H0: open condition, L0: shade condition (relative irradiance of ≈ 13% of open condition) throughout the experimental period, LH1: transfer from shade to open on Oct 5, LH2: transfer from shade to open on Oct 12, and LH3: transfer from shade to open on Oct 18. Values are means ± se; n= 3 to 6 for attached leaves, sampled from Aug 2 to Oct 25. * denotes significant effect at P ≤ 0.05, ** P ≤ 0.01, and *** P ≤ 0.001. ns indicates no significant effect. Different letters indicate significant differences among means of the light treatments on the same date at P < 0.05.

Leaf starch content decreased from mid-summer to autumn, and reached almost 0 before shedding, irrespective of growth light conditions (**Figures 4B, D**). On 6^th^ Oct, one day after the transfer of LH1 seedlings to the open condition, significantly higher leaf starch content was observed in LH1 seedlings compared with L0 seedlings (**Figure 4D**; **Table 4**).

**Table 4:** Seasonal change in dry-mass based leaf starch content (mg g^-1^) in leaves of fullmoon maple seedlings grown under different light conditions.

Date	Open	Shade				
	H0	L0	LH1	LH2	LH3	F-statistics
Aug 2	14.6 ± 2.6	11.2 ± 3.2	―	―	―	0.66^ns^ (F_1,6_)
Aug 20	22.3 ± 3.4	18.4 ± 5.7	―	―	―	0.36^ns^ (F_1,6_)
Sep 8	13.7 ± 1.9	5.3 ± 4.3	―	―	―	3.04^ns^ (F_1,6_)
Sep 29	7.3 ± 1.5	8.8 ± 1.8	―	―	―	0.40^ns^ (F_1,6_)
Oct 6	7.9 ± 0.5 ^ab^	4.5 ± 0.8 ^b^	10.4 ± 1.7 ^a^	―	―	8.62^*^ (F_2,8_)
Oct 13	5.8 ± 2.0	4.3 ± 0.6	8.3 ± 3.9	6.8 ± 1.5	―	0.64^ns^ (F_3,11_)
Oct 19	6.6 ± 2.1	2.6 ± 0.8	5.3 ± 3.7	2.5 ± 1.2	4.5 ± 1.6	1.00^ns^ (F_4,17_)
Oct 25	1.9 ± 0.8 ^a^	0.3 ± 0.3	0.4 ± 0.7	0.5 ± 0.4	0.0 ± 0.3	2.08^ns^ (F_4,15_)

H0: open condition, L0: shade condition (relative irradiance of ≈ 13% of open condition) throughout the experimental period, LH1: transfer from shade to open on Oct 5, LH2: transfer from shade to open on Oct 12, and LH3: transfer from shade to open on Oct 18. Values are means ± se; n= 3 to 6 for attached leaves, sampled from Aug 2 to Oct 25. * denotes significant effect at P ≤ 0.05. ns indicates no significant effect. Different letters indicate significant differences among means of light treatments on the same date at P < 0.05.

**Figure 5:**
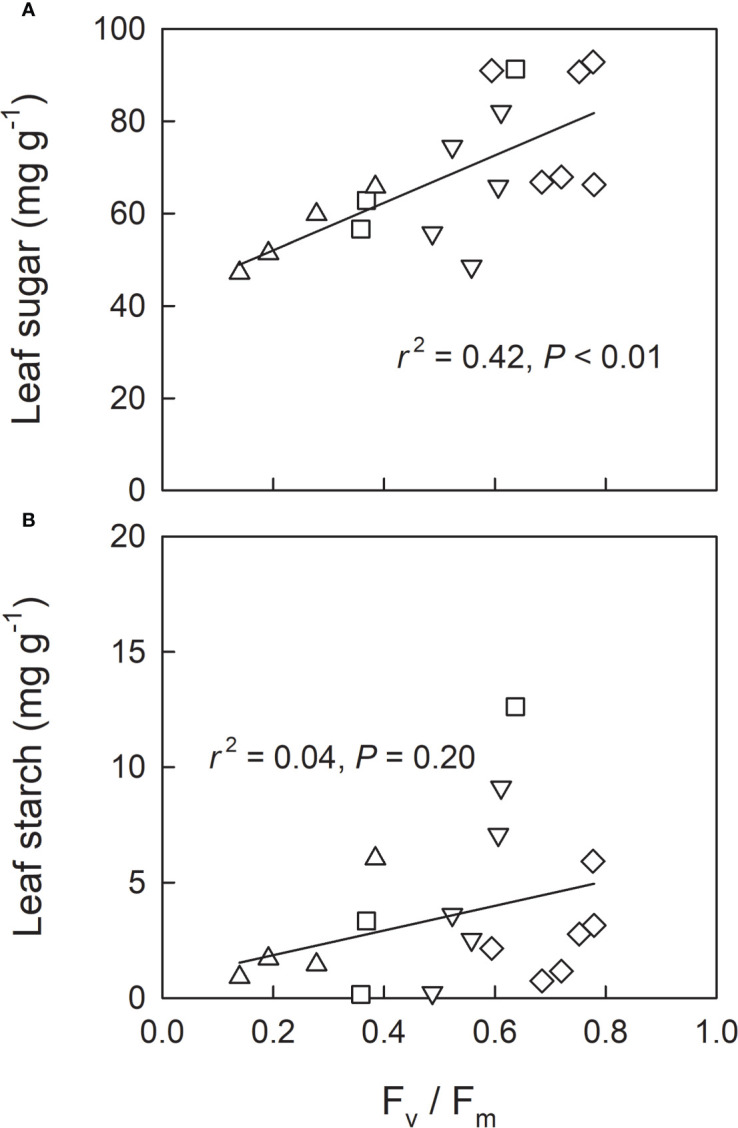
Relationship between F_v_/F_m_ and leaf soluble sugar content **(A)** and leaf starch content **(B)** in leaves (measured and sampled on 19^th^ Oct) of fullmoon maple seedlings grown under shade (L0, diamond), transferred from shade to open conditions on 5^th^ Oct (LH1, square), on 12^th^ Oct (LH2, triangle-up), and on 18^th^ Oct (LH3, triangle-down). Linear regression analysis was conducted for the pooled data across the light treatments.

**Figure 6:**
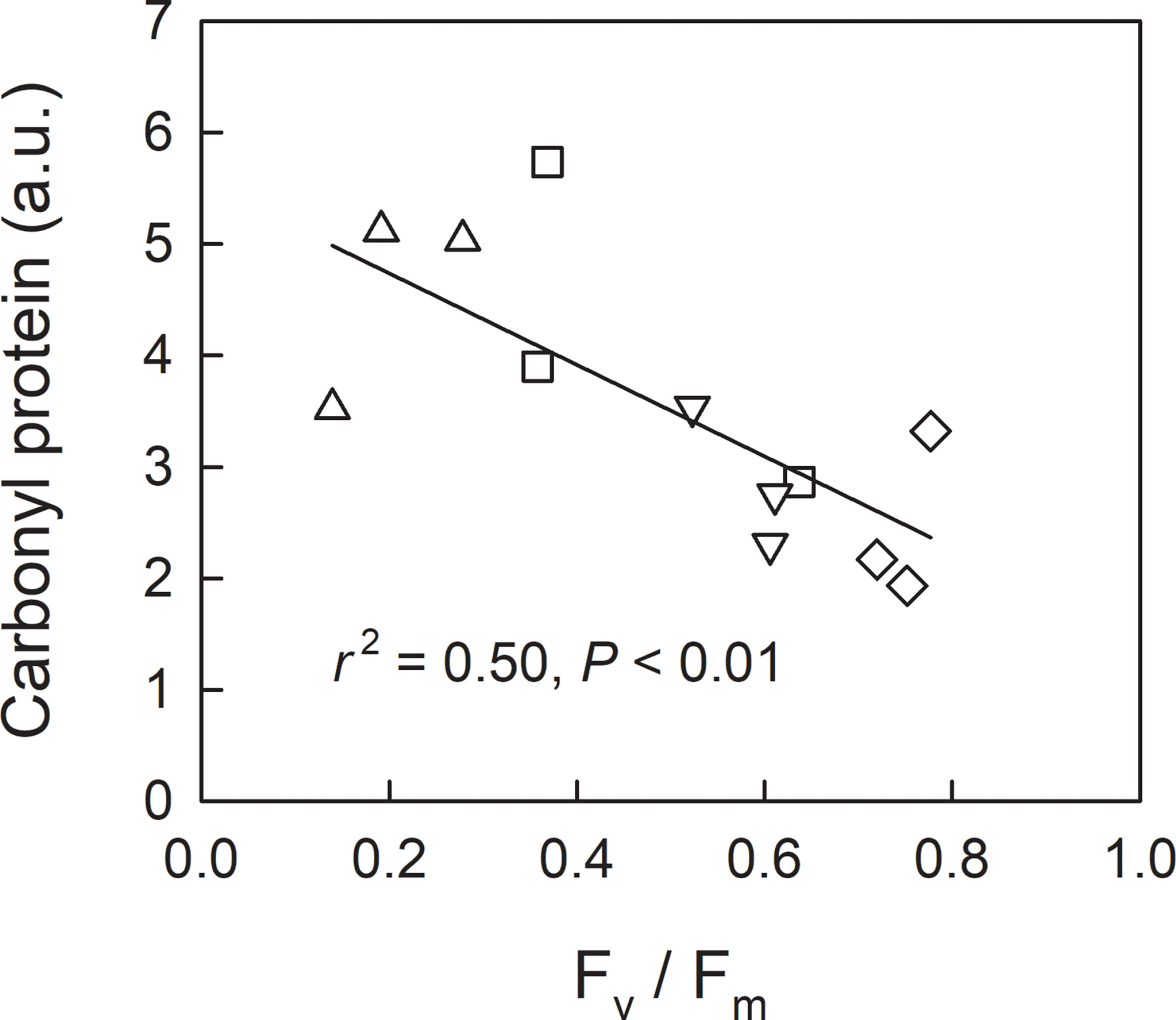
Relationship between F_v_/F_m_ and carbonyl protein in leaves (measured and sampled on 19^th^ Oct) of fullmoon maple seedlings grown under shade (L0, diamond), transferred from shade to open conditions on 5^th^ Oct (LH1, square), 12^th^ Oct (LH2, triangle-up), and 18^th^ Oct (LH3, triangle-down). The amount of carbonyl protein was expressed in an arbitrary unit (a.u.). Linear regression analysis was conducted for the pooled data across the light treatments.

We investigated leaf sugar and starch as well as protein carbonylation in shade-grown seedlings (L0, LH1, LH2, and LH3) as a function of F_v_/F_m_ on 19^th^ Oct, when approximately half of the retrievable N had been resorbed (**Figure 3D**). Across the treatments, leaf sugar content decreased with decreasing F_v_/F_m_ (**Figure 5A**), whereas no significant linear relationship between leaf starch and F_v_/F_m_ was observed (**Figure 5B**). Conversely, protein carbonylation, which is a good indicator of oxidative stress by reactive oxygen species (ROS) (Anjum et al., 2015), increased with decreasing F_v_/F_m_ (**Figure 6**).

Dry mass-based leaf chlorophyll content (Chl a+b) was lower in open-grown seedlings (H0) than in fully-shade-grown seedlings (L0) (**Table 5**). However, as leaf mass per area was higher in open-grown seedlings (H0) than shade-grown seedlings (L0, LH1, LH2, and LH3), area-based leaf chlorophyll was not significantly different among the light treatments (data not shown). The ratio of chlorophyll a to b (Chl a:b) was not significantly different among the light treatments. Chlorophyll-based xanthophyll cycle pool size (sum of xanthophyll pigments: violaxanthin, antheraxanthin and zeaxanthin) ((V+A+Z)/Chl) was significantly higher in H0 seedlings than shade-grown seedlings (L0, LH1, LH2, and LH3). The conversion state of xanthophyll cycle (Z+A)/(V+A+Z) showed higher values in H0, LH1, LH2, and LH3 leaves sampled under full sunlight than in L0 leaves sampled under shade. Chlorophyll-based anthocyanins (anthocyanin/Chl) was higher in open-grown (H0) seedlings, than in shade-grown (L0), and 1 day open-exposed (LH3) seedlings.

**Table 5:** Leaf mass per area (LMA) and pigments in leaves of fullmoon maple seedlings grown under different light conditions, sampled in the morning of Oct 19.

Oct 19	Open	Shade				
	H0	L0	LH1	LH2	LH3	F-statistics
LMA (g m^-2^)	62.5 ± 3.6 ^a^	35.4 ± 1.2 ^b^	37.0 ± 1.8 ^b^	34.4 ± 0.7 ^b^	39.4 ± 3.0 ^b^	23.4 ^***^ (F_4,17_)
Chl a+b (µmol g^-1^)	2.98 ± 0.39 ^b^	8.55 ± 1.06 ^a^	6.70 ± 0.32 ^ab^	6.54 ± 0.67 ^ab^	5.87 ± 1.17 ^ab^	4.63 ^*^ (F_4,17_)
Chl a:b	2.55 ± 0.09	2.65 ± 0.14	2.21 ± 0.12	2.38 ± 0.12	2.65 ± 0.07	2.30 ^ns^ (F_4,17_)
(V+A+Z)/Chl (mmol mol^-1^)	154.2 ± 16.9 ^a^	57.7 ± 9.4 ^b^	94.2 ± 5.1 ^b^	88.5 ± 11.6 ^b^	69.0 ± 11.9 ^b^	9.96 ^***^ (F_4,17_)
(Z+A)/(V+A+Z)	0.79 ± 0.05 ^a^	0.57 ± 0.06 ^b^	0.88 ± 0.02 ^a^	0.81 ± 0.03 ^a^	0.78 ± 0.02 ^a^	6.76 ^**^ (F_4,17_)
Anthocyanin/Chl (mol mol^-1^)	2.32 ± 0.65 ^a^	0.63 ± 0.34 ^b^	1.87 ± 0.10 ^ab^	0.91 ± 0.19 ^ab^	0.58 ± 0.29 ^b^	4.14 ^*^ (F_4,17_)

Pigments analyzed were dry-mass based leaf total chlorophyll content (Chl a+b), chlorophyll a to b ratio (Chl a:b), chlorophyll-based xanthophyll cycle pigments content ((V+A+Z)/Chl), including violaxanthin (V), antheraxanthin (A), and zeaxanthin (Z), conversion state of xanthophyll cycle ((Z+A)/(V+A+Z)), chlorophyll-based anthocyanins content (anthocyanin/Chl). H0: open condition, L0: shade condition (relative irradiance of ≈ 13% of open condition) throughout the experimental period, LH1: transfer from shade to open on Oct 5, LH2: transfer from shade to open on Oct 12, and LH3: transfer from shade to open on Oct 18. Values are means ± se; n= 3 to 6. *, **, and *** denote significant effect at P ≤ 0.05, ≤ 0.01, and ≤ 0.001, respectively. ns indicates no statistically significant effect. Different letters indicate significant differences among means of light treatments on the same date at P < 0.05.

## Discussion

In the present study, early shedding of outer canopy leaves, simulated by transferring shade-grown seedlings to fully-open light-exposed conditions, caused significantly lower N resorption in the seedlings transferred on 12^th^ Oct (LH2). Leaves of LH2 seedlings showed a significantly lower F_v_/F_m_ compared to L0, LH1, and LH3 on 19^th^ Oct, when approximately half of resorbable N had been exported from the leaves in all treatments. Across the treatments, higher degree of protein carbonylation was observed in leaves with lower F_v_/F_m_ on 19^th^ Oct. This suggests that photoinhibition, indicated by a decrease in F_v_/F_m_ (Krause, 1994; Werner et al., 2002), might be closely-associated with oxidative stress by ROS, indicated by protein carbonylation (Anjum et al., 2015). Leaf sugar accumulation might be necessary for an efficient N resorption as a driving energy for chlorophyll and protein catabolism (Hörtensteiner and Feller, 2002), and amino acids export (Liu et al., 2008; Okumoto and Pilot, 2011), as well as for a regulative signal of leaf senescence under low temperature (Stitt and Hurry, 2002; Wingler et al., 2006; Tarkowski and Van den Ende, 2015). Shade-grown seedlings (L0) increased leaf sugar content to a level comparable to sun-grown seedlings (H0) in autumn, also suggesting the relevance of leaf sugar accumulation for leaf N resorption. In this context, smaller sugar content in LH2 seedlings with severer photoinhibition, indicated by the lower F_v_/F_m_ on 19^th^ Oct (Krause, 1994; Werner et al., 2002), might result in lower N resorption efficiency.

An increase in protein carbonylation is a biomarker of oxidative stress by ROS (Anjum et al., 2015). A greater amount of protein carbonylation was observed with decreasing F_v_/F_m_, suggesting that oxidative damage of proteins facilitated by ROS might occur in shade-grown seedlings transferred into open condition, especially in LH2 seedlings. Oxidative stress, indicated by an increase in protein carbonylation, might also increase the risk of membrane peroxidation (Anjum et al., 2015). Membrane intactness is also important for recycling N from the photosynthetic apparatus (Hörtensteiner and Feller, 2002) as well as for amino acid export (Okumoto and Pilot, 2011). Membrane peroxidation might be a cause of the insufficient N resorption observed in LH2 seedlings. It is noteworthy that an involvement of ROS in Rubisco degradation (Desimone et al., 1996; Stieger and Feller, 1997; Ishida et al., 1998; Hörtensteiner and Feller, 2002) might be reflected on the later start of N resorption in shade-grown seedlings (L0) compared with open-grown seedlings (H0), as well as the retarded N resorption in L0 seedlings compared with LH1 and LH3 on 25^th^ Oct.

Higher LMA is a typical morphological trait in sun leaves, which also have higher area-based photosynthetic capacity (2018b; Niinemets et al., 2004; Kitao et al., 2006). In the present study, open and shade treatments were appropriate to allow the study of typical sun and shade leaves (**Table 5**). Assuming that anthocyanins have a photoprotective role as light attenuators and antioxidants (Neill et al., 2002; Moustaka et al., 2020), open-grown seedlings (H0) had higher photoprotective capacity by anthocyanins than shade-grown (L0) and 1 day open-exposed seedlings (LH3). Moreover, LH1 (14 days open-exposed), and LH2 (7 days open-exposed) seedlings showed intermediate values of anthocyanins between H0, and L0 and LH3, suggesting that anthocyanins might gradually accumulate at a time scale of a few days. The conversion state of xanthophyll cycle [(Z+A)/(V+A+Z)], which is closely related to thermal energy dissipation (Demmig-Adams and Adams, 1992; Verhoeven et al., 1999), promptly responded to the increase in solar radiation, where LH1, LH2, and even LH3 (1 day after the transfer) seedlings showed a comparable value of [(Z+A)/(V+A+Z)] to that in H0 seedlings grown under full sunlight. Although the pool size of xanthophyll cycle was different between open-grown seedlings (H0) and shade-grown seedlings (L0, LH1, LH2, and LH3), conversion of xanthophyll pigments to (Z+A) was well functioned against high light in the open-transferred seedlings (LH1, LH2, and LH3). Some photoprotective responses such as increases in anthocyanins, and [(Z+A)/(V+A+Z)], compared to shade-grown L0 seedlings, were observed in LH1 and LH2 seedlings. As F_v_/F_m_ in LH1 on 19^th^ Oct was comparable to F_v_/F_m_ in open-grown H0 seedlings (**Table 1**), LH1 seedlings might have successfully acclimated to high light condition. Conversely, the photoprotective responses might not be sufficient enough in LH2 seedlings since significantly lower F_v_/F_m_ on 19^th^ Oct was observed, compared to H0 seedlings.

The higher starch content observed in LH1 than in L0 on 6^th^ Oct, one day after the transfer, suggests a positive effect of transfer into open condition with greater amount of solar radiation on the photosynthetic carbon gain, in spite of apparent photoinhibition. LH1 seedlings also showed a temporal cessation of photoinhibition from 6^th^ to 13^th^ Oct, and comparable F_v_/F_m_ to that in H0 on 19^th^ Oct, with accumulation of anthocyanins and increased xanthophyll cycle conversion, suggesting acclimation to the high light condition (Tobita et al., 2010). This acclimation might result from the relatively high air temperature around 15°C at the first week after the seedling transfer, and the moderate initial impact of solar radiation (PAR_int_ on the first day of transfer: 11.0 mol m^-2^ day^-1^). Conversely, LH2 seedlings were exposed to substantially high PAR_int_ (≈25 mol m^-2^ day^-1^) on the first three days after transfer, and suffered low temperatures down to 5°C in the following week, which might induce the most severe photoinhibition with incomplete acclimation (Ball, 1994; Krause, 1994). Regarding LH3 seedlings, the initial PAR_int_ was intermediate (15.5 mol m^-2^ day^-1^), but the air temperature was relatively low (below 10°C), leading to fast-progressing photoinhibition. However, the extent of photoinhibition in LH3 seedlings was limited on 19^th^ Oct because of the shorter period (1 day) of exposure to open radiation. Although the degree of photoinhibition seems to be dependent on solar radiation and air temperature as well as the timing of simulated leaf shedding (2018a; 2022; Werner et al., 2001; Kitao et al., 2004), severer photoinhibition, as was observed in LH2, might increase the risk of incomplete N resorption by oxidative damage.

Based on the findings described above, we propose the “holocanopy hypothesis” to describe the phenomenon where the outer-canopy leaves of fullmoon maple, the longevity of which is prolonged with anthocyanins (2003; Feild et al., 2001; Hoch et al., 2001; Schaberg et al., 2008; Lo Piccolo et al., 2018; Moustaka et al., 2020; Lo Piccolo and Landi, 2021), protect inner-canopy leaves from photooxidative stress by shading, contributing to efficient N resorption as a whole canopy. This hypothesis deserves further experimentation and validation in different species and experimental systems. Based on field observations, the timing of leaf shedding is quite synchronized in all leaves within a canopy of fullmoon maple, as leaf age of fullmoon maple is similar due to flush-type shoot development (Kikuzawa, 1983; Koike, 1990). In the case of synchronized leaf senescence, light attenuation by the outer-canopy leaves would be of relevance to protect the inner-canopy leaves against photooxidative stress during leaf N resorption. Although further investigation in other autumn-coloring species is needed, whole-canopy responses warrant consideration to understand the role of autumn coloring.

## Data availability statement

The original contributions presented in the study are included in the article/**Supplementary Material**. Further inquiries can be directed to the corresponding author.

## Author contributions

MK: Conceptualization, Investigation, Formal analysis, Writing - Original Draft. KY: Investigation, Writing - Review & Editing. HT: Investigation, Writing - Review & Editing. EA: Validation, Writing - Review & Editing. JK: Investigation, Writing - Review & Editing. AT: Investigation, Writing - Review & Editing. RT: Conceptualization, Investigation, Writing - Review & Editing. All authors contributed to the article and approved the submitted version.

## Funding

This study was supported in part by JSPS KAKENHI Grant Number JP20H03036 and JP20H03017 to MK and RT. EA also acknowledges support by the National Natural Science Foundation of China (No. 4210070867).

## Acknowledgments

We thank H. Yamamoto for her skillful technical assistance in cultivating the pot-grown seedlings.

## Conflict of interest

The authors declare that the research was conducted in the absence of any commercial or financial relationships that could be construed as a potential conflict of interest.

## Publisher’s note

All claims expressed in this article are solely those of the authors and do not necessarily represent those of their affiliated organizations, or those of the publisher, the editors and the reviewers. Any product that may be evaluated in this article, or claim that may be made by its manufacturer, is not guaranteed or endorsed by the publisher.
