# Supplementary material for: Exposure to strong irradiance exacerbates photoinhibition and suppresses N resorption during leaf senescence in shade-grown seedlings of fullmoon maple (Acer japonicum)
Source: Front Plant Sci. 2022 Oct 28;13:1006413. doi: 10.3389/fpls.2022.1006413 (PMC9650427; doi:10.3389/fpls.2022.1006413)
Supplement: Supplementary file 1 [file Image_1.pdf]

# *Supplementary Material*

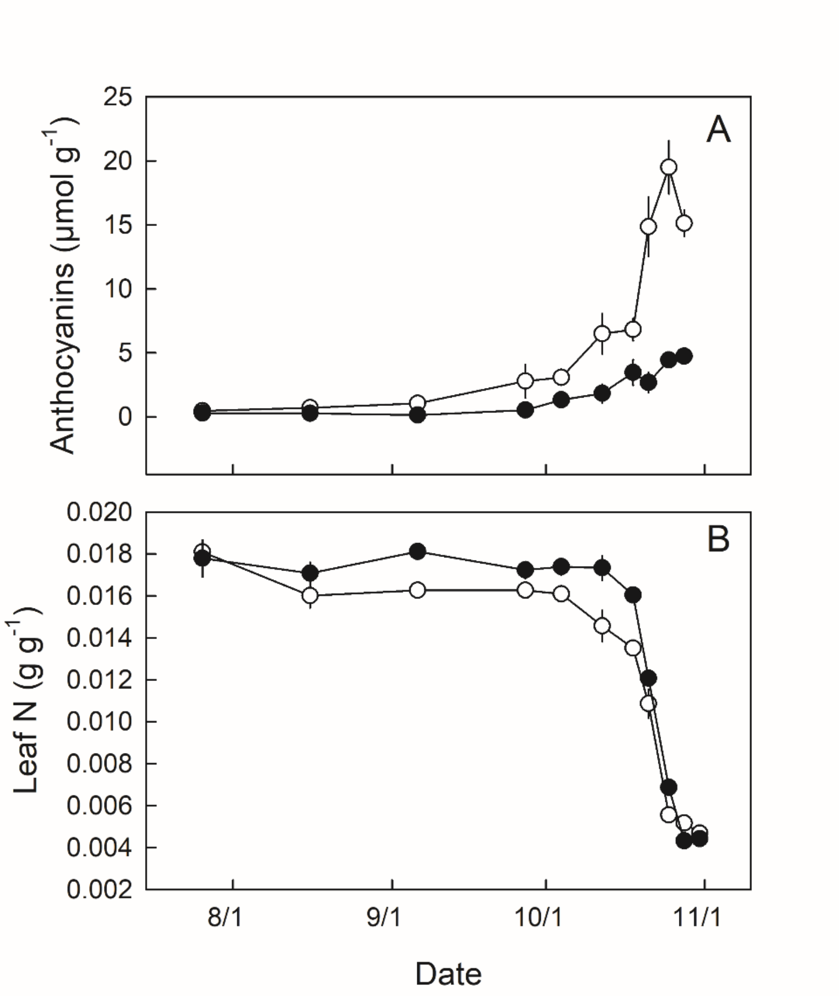


**Supplementary Figure 1.** Seasonal changes in dry mass-based leaf anthocyanins content (A), and leaf N content (B) in the outer- (open) and the inner-canopy (closed symbols) leaves of fullmoon maple. Leaves were sampled from an adult tree of fullmoon maple (height: ≈10 m, age: 50 years old) grown in the arboretum of Hokkaido Research Center, Forestry and Forest Products Research Institute (43.0°N, 141.4°E; 180 m a.s.l.). Outer-canopy leaves had higher amount of anthocyanins than inner-canopy leaves during leaf senescence, whereas leaf senescence progressed synchronously. Values are mean ± se (n=4).
